# Functional excitation-inhibition ratio for social anxiety analysis and severity assessment

**DOI:** 10.3389/fpsyt.2024.1461290

**Published:** 2024-10-22

**Authors:** Linh Ha Chu, Chi Que Chau, Nidal Kamel, Huong Ha Thi Thanh, Norashikin Yahya

**Affiliations:** ^1^ College of Engineering and Computer Science, Center of Environmental Intelligence (CEI), Vin University, Hanoi, Vietnam; ^2^ Department of Tissue Engineering and Regenerative Medicine, School of Biomedical Engineering, International University, Vietnam National University, Ho Chi Minh City, Vietnam; ^3^ Department of Electrical and Electronics Engineering, Universiti Teknologi PETRONAS (UTP), Bandar Seri Iskandar, Malaysia

**Keywords:** social anxiety disorder (SAD), EEG, excitation inhibition balance, resting state network, default mode network (DMN)

## Abstract

**Introduction:**

Social anxiety disorder (SAD) is a prevalent psychiatric condition characterized by an intense fear of and avoidance of social situations. Traditional assessment methods for SAD primarily rely on subjective self-report questionnaires and clinical interviews, which can be prone to biases and inaccuracies. This study aims to explore the functional excitation-inhibition (fEI) ratio derived from EEG data as a potential objective biomarker for assessing SAD severity.

**Methods:**

Resting-state EEG data were collected from 20 control subjects and 60 individuals with varying degrees of SAD severity (mild, moderate, and severe). The fEI ratio was estimated across different EEG bands and analyzed, focusing on differences between control subjects and SAD groups.

**Results:**

Significantly higher fEI ratios were observed in the alpha and low beta EEG bands in individuals with SAD compared to controls, especially within the prefrontal cortex. Additionally, a positive correlation was found between the fEI ratio and the severity of social anxiety symptoms across SAD severity levels.

**Discussion:**

The findings indicate that the fEI ratio in the alpha and low beta bands may serve as a promising biomarker for assessing SAD severity. These results contribute to a deeper understanding of the neural mechanisms underlying social anxiety, offering a potentially more objective approach to SAD assessment compared to traditional methods.

## 1 Introduction

Social Anxiety Disorder (SAD) is a prevalent psychiatric condition marked by an intense fear and avoidance of social situations due to perceived scrutiny from others (1). It significantly impairs various aspects of individuals’ lives, reducing their quality of life (2) (3–5). Despite its prevalence and impact, accurately detecting and assessing SAD remains challenging, as current methods rely heavily on subjective self-report measures and clinical interviews, which are susceptible to biases and inaccuracies (6).

Recently, there has been growing interest in using neuroimaging techniques, such as Electroencephalography (EEG), to better understand the neural mechanisms underlying SAD and develop objective biomarkers for its detection and severity assessment. EEG provides a direct measure of brain activity with high temporal resolution, making it a promising tool for studying the dynamic neural processes associated with SAD.

Anxiety disorders, including SAD, are linked to alterations in the activity and connectivity of specific brain regions involved in emotion regulation, threat processing, and social cognition (7). Neuroimaging studies using techniques like functional magnetic resonance imaging (fMRI) and EEG have identified key brain regions implicated in anxiety, such as the amygdala, prefrontal cortex, insula, and anterior cingulate cortex (8). The amygdala, a crucial structure in the brain’s limbic system, plays a central role in processing fear and threat-related stimuli (9), and hyperactivity in this region is commonly observed in individuals with anxiety disorders (10). The prefrontal cortex, particularly the ventromedial and dorsolateral prefrontal cortices, is involved in emotion regulation and cognitive control processes (11), with dysfunction in these areas associated with heightened anxiety responses (12). The insula is implicated in interoceptive awareness and the experience of bodily sensations (13), while the anterior cingulate cortex is involved in monitoring and regulating emotional responses (14). Dysregulation of these brain regions and their interconnected networks likely contributes to the excessive fear and avoidance behaviors characteristic of anxiety disorders (15).

Resting-state EEG has been used to investigate the relationship between brain activity patterns and anxiety (16–18). Studies have shown that individuals with anxiety disorders exhibit alterations in resting-state brain wave patterns compared to healthy individuals (19). These abnormal patterns may reflect heightened arousal, altered information processing, or difficulty in regulating emotions. Frontal alpha asymmetry, a specific pattern of EEG activity in the frontal brain regions, has been linked to anxiety (20). Greater relative right frontal activity (i.e., higher alpha power in the right frontal region compared to the left) is associated with negative affect and anxiety (21). Resting-state EEG also allows researchers to examine functional connectivity patterns between different brain regions in the absence of specific tasks or stimuli (19, 22). Studies have found alterations in functional connectivity within the default mode network (DMN) (17), salience network (23), and other brain networks in individuals with anxiety disorders (24).

The excitation-inhibition (E/I) ratio has emerged as a critical biomarker in understanding the neural mechanisms underlying various psychiatric conditions, including Generalized Anxiety Disorder (GAD), Major Depressive Disorder (MDD), and Seasonal Affective Disorder (SAD). In GAD, an imbalance characterized by increased excitatory and decreased inhibitory neurotransmission has been linked to hyperactivity in brain regions such as the amygdala and prefrontal cortex, demonstrating high sensitivity in detecting anxiety-related neural changes (25). Clinical studies suggest that treatments like cognitive-behavioral therapy and pharmacotherapy can restore E/I balance, indicating its potential as a marker for monitoring treatment responses (26). Similarly, in MDD, alterations in the E/I ratio, often reflecting increased inhibition in the prefrontal cortex and hippocampus, contribute to symptoms such as anhedonia and cognitive deficits. The ratio’s sensitivity is confirmed by consistent findings across neuroimaging and electrophysiological studies (27). Moreover, therapeutic interventions like antidepressants and electroconvulsive therapy (ECT) have been shown to normalize the E/I ratio, correlating with clinical improvements (28). In SAD, seasonal changes in light exposure impact the E/I ratio, influencing mood and behavior, particularly during winter months. While research is ongoing, early findings suggest that the E/I ratio could be vital for understanding and treating seasonal mood fluctuations, with light therapy playing a key role in modulating this balance (29–31). Overall, the E/I ratio’s sensitivity, validity, and clinical utility across these disorders highlight its potential as a diagnostic and therapeutic tool, warranting further investigation into its broader applications in personalized medicine.

Recent studies indicate that the functional excitation-inhibition (fEI) ratio in resting-state EEG plays a crucial role in anxiety disorders (32). Imbalances in the fEI ratio can contribute to the development and maintenance of anxiety disorders. For instance, an imbalance between excitation and inhibition in brain regions such as the amygdala and prefrontal cortex can lead to hyperactivity in neural circuits involved in emotion regulation and fear responses (33). This imbalance can impair fear extinction mechanisms, leading to persistent and exaggerated fear responses (34). Additionally, neurotransmitters such as gamma-aminobutyric acid (GABA) and glutamate play key roles in regulating the fEI balance (35). Dysregulation of these neurotransmitter systems can disrupt the balance between excitation and inhibition, contributing to anxiety disorders.

In this paper, we present a novel application of the fEI ratio metric to resting-state EEG data for the detection and severity assessment of SAD. We hypothesize that aberrant patterns of excitation-inhibition balance in specific brain regions may distinguish individuals with SAD from healthy controls and correlate with the severity of their symptoms. By investigating the neural correlates of SAD using the fEI ratio metric, we aim to enhance our understanding of the neurobiological mechanisms underlying this disorder and pave the way for the development of objective biomarkers for its diagnosis and treatment monitoring. Understanding the implications of the fEI ratio in anxiety disorders is crucial for developing novel therapeutic approaches targeting the underlying neurobiological mechanisms.

This paper is organized as follows: the first section provides an overview of the research topic and a review of relevant literature to provide context and background for the study. The second section outlines the methodology used to conduct the research, including data collection methods and analysis techniques. The third section presents the findings of the study, while the fourth section discusses the implications and significance of these findings. Finally, the last section offers conclusions, recommendations, and suggestions for future research in this area. By following this structured organization, this paper aims to provide a comprehensive and coherent exploration of the research topic.

## 2 Experimental setup

### 2.1 Participants

Eighty-nine participants were recruited from 502 respondents who completed the Social Interaction Anxiety Scale (SIAS) self-assessment reports. To ensure the findings of our study were robust and generalizable, both genders were included in the experiment. The severity of SAD was assessed for each participant using the SIAS scale.

The Social Interaction Anxiety Scale (SIAS) is a widely used self-report tool developed by Mattick and Clarke in 1998 to assess social anxiety, particularly the distress experienced during social interactions (36). It consists of 20 items rated on a Likert scale, with higher scores indicating greater social anxiety. The SIAS is highly specific to social interaction anxiety, demonstrating strong reliability, validity, and ease of use, making it a valuable tool in both clinical and research settings. Scores on the SIAS can classify social anxiety severity into mild, moderate, and severe, aiding in diagnosis and treatment planning for Social Anxiety Disorder (SAD). Its robust psychometric properties, including high internal consistency and test-retest reliability, support its utility as a diagnostic tool aligned with SAD criteria as outlined in the DSM-5 (37, 38).

Participants were categorized into four groups based on their SIAS scores: healthy controls (HC) with scores below 20, mild SAD with scores below 40, moderate SAD with scores below 60, and severe SAD with scores of 60 or above. One participant was excluded due to data acquisition issues. Age did not show significant differences between the groups, F(1, 87) = 2.664, p = 0.054, η² = 0.093. All participants were right-handed to generalize hemisphere dominance, and all were mentally and physically healthy with no signs of brain damage. There was no history of neurological, psychiatric, or surgical disorders among the participants that could have affected metabolic or brain function. During the EEG session recruitment process, none of the subjects were undergoing pharmacological or psychotherapeutic treatment. All participants reported having normal or corrected-to-normal vision based on their self-reports.

In line with the CONSORT guidelines, a power analysis was conducted prior to the study to justify the selected sample size. The analysis was based on expected effect sizes from similar studies in the field, aiming for a target power of 80% and an alpha level of 0.05. This ensured the sample size was sufficient to detect meaningful differences between groups while accounting for variability in the data.

Each selected participant received a single page with all study data, a waiver of written informed consent, and an honorarium to compensate for their time and cooperation. The demographic information and participant characteristics are detailed in **Table 1**. The protocol for the study was carefully reviewed, accepted, and approved by the Medical Research Ethics Committee of the Royal College of Medicine Perak, Kuala Lumpur University, Malaysia.

**Table 1:** Demographic data and group characteristics (22).

Group	Number of participants		Total	Age		SIAS score	
	Female	Male		Female	Male	Female	Male
Severe	12	10	22	22.13 ± 2.78	23.11 ± 1.02	67.53 ± 6.21	66.81 ± 5.32
Moderate	7	15	22	21.98 ± 3.11	22.21 ± 1.25	55.73 ± 7.81	54.41 ± 6.61
Mild	12	10	22	22.61 ± 2.32	21.71 ± 2.31	38.32 ± 5.12	37.71 ± 5.81
Control	8	14	22	21.76 ± 1.73	23.62 ± 1.65	14.71 ± 6.74	16.61 ± 7.34

### 2.2 EEG preprocessing

**Figure 1:**
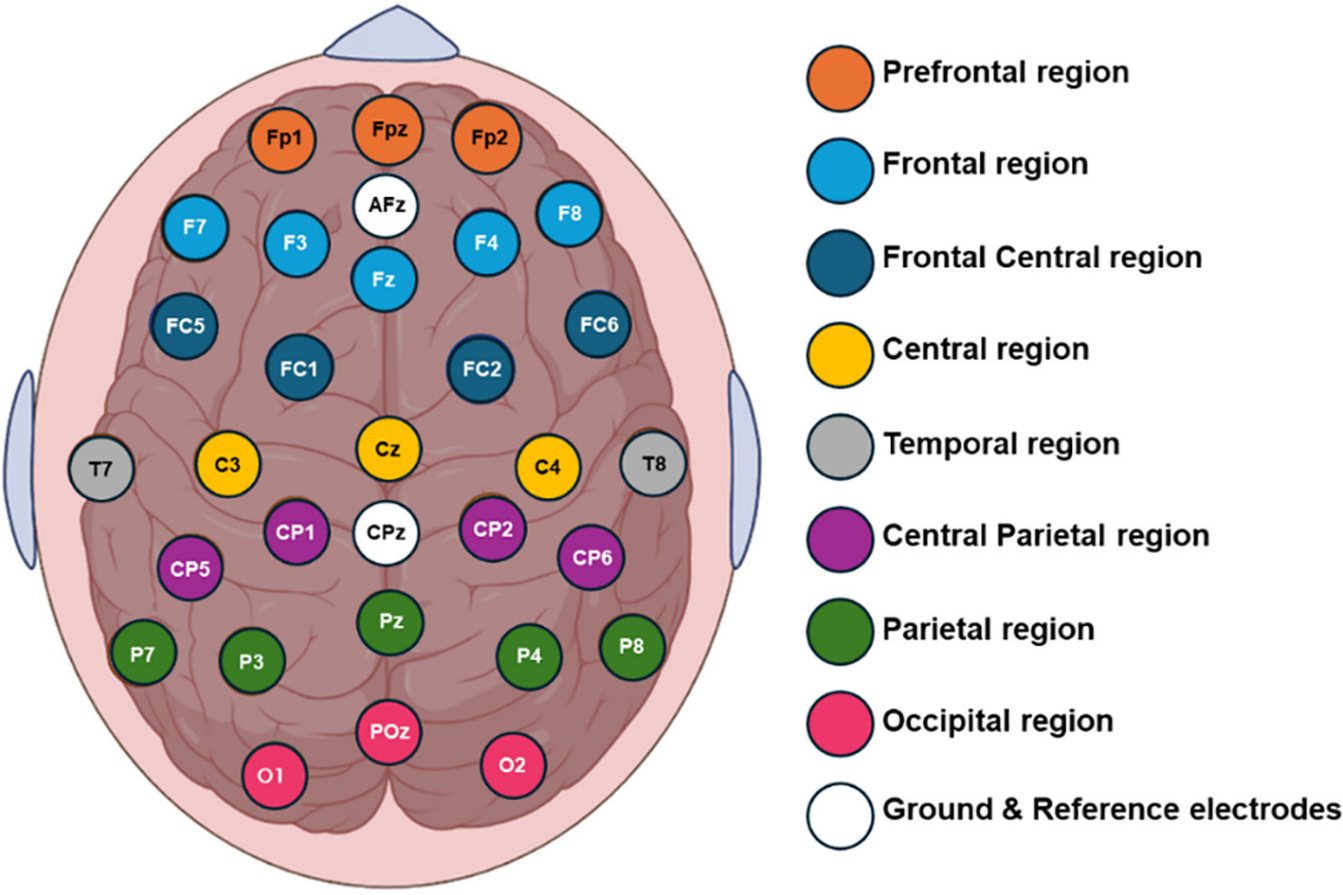
Topographical placement of 32 electrodes using the extended 10-20 international system, indicating the distribution of the electrodes on the cortical scalp.

Six-minute baseline real-time EEG data were recorded using a 32-channel shielded cap (ANT Neuro, Enschede, Netherlands). The cap included 32 gel-based sensors mounted on the scalp, grounded at AFz, and referenced to CPz. The recorded EEG signals were then re-referenced to a common average reference. Impedance was maintained below 10 kΩ. The EEG signals were initially sampled at 2048 Hz and subsequently down sampled to 256 Hz. **Figure 1** illustrates the topographic distribution of EEG electrodes across various cortical regions: prefrontal (Fp1, Fpz, Fp2), frontal (F7, F3, Fz, F4, F8), frontal central (FC5, FC1, FC2, FC6), central (C3, Cz, C4), temporal (T7, T8), central parietal (CP5, CP1, CPz, CP2, CP6), parietal (P7, P3, P4, P8), and occipital (O1, POz, O2). To isolate the signal segment within the 0.4 to 50 Hz frequency range, an FIR band-pass filter was applied to remove noise, high-frequency artifacts, and low-frequency distortions. EEG signals were obtained from multiple active sensors positioned in a consistent spatial configuration adhering to international standards. Artifacts such as horizontal (HEOG) and vertical (VEOG) eye movements, eye blinks, breathing, cardiac movements, and power interference were visually inspected and automatically discarded using spatial filters for artifact detection and correction provided by BESA software. Additionally, the open-source toolbox EEGLAB was utilized for visualizing the topographic maps (39).

### 2.3 The functional excitation-inhibition ratio calculation

To estimate the *f*EI ratio, it is essential to establish a covariation between the oscillation amplitude and the long-range temporal correlations (LRTC). To verify this requirement, we perform detrended fluctuation analysis (DFA) on the data, ensuring the existence of LRTC with a threshold DFA exponent greater than 0.55. This analysis is conducted for signals in the alpha (8-12 Hz) and low-beta (13-18 Hz) frequency bands.

After filtering the signal, we calculate the amplitude envelope using the absolute Hilbert Transform. The resulting signal is then used to calculate the signal profile, defined as the cumulative sum of the demeaned amplitude envelope. For DFA analysis, the signal profile is divided into windows of specific sizes. During the DFA calculation, the fluctuation function is plotted on a logarithmic scale, with window sizes ranging from 5 to 12 seconds and a 50% overlap. Each window size is equally spaced on the logarithmic time scale.

Prior to detrending, the signal in each signal profile window is normalized by dividing it by the mean of the amplitude envelope within that window. The DFA exponent is then calculated as the slope of the fluctuation function, as follows (40):

Compute the cumulative sum of the time series to create the signal profile.

1. Divide the signal profile into a set, W , of 50% overlapped windows of size t .
2. Remove the linear trend (using a least-squares fit) from the time series in each window, *w* ^detrend^.
3. Calculate the standard deviation of the detrended signal within each window, σ(*w* ^detrend^).
4. Compute fluctuation function as the mean standard deviation of all the windows.
〈F(t)〉 =mean(σ(W))
5. Using linear regression, the DFA exponent is estimated as the slope of the trend line of the fluctuation function.

If the DFA exponent exceeds the threshold, the normalized fluctuation function, nF(t) estimates LRTC on a short-time scale, is calculated by dividing each window by its original amplitude.

Subsequently, the *f*EI ratio is calculated as follows (40):


$$fEI\;=\;1-\;r_{W_{amp},\;\;W_{nF\left(t\right)}}$$

where $r_{W_{amp},\;W_{nF\left(t\right)}\;}$ is the Pearson correlation between the set of detrended windowed amplitude-normalized signal profiles ( $W_{nF\left(t\right)}$ ) and the set of windowed amplitude values ( $W_{amp}$ ). With this calculation, the network that is inhibition-dominated will have an *f*EI below 1, the network with excitation-dominated will have an *f*EI ratio greater than 1, and a critical network will have an *f*EI of 1.

We checked for outliers using standardized residuals and Cook’s Distance and assessed multicollinearity through Variance Inflation Factors (VIF), finding no issues with values exceeding the threshold of 5. To control for multiple comparisons, we applied Bonferroni correction after ANOVA and used Tukey’s HSD for *post-hoc* pairwise tests. ANOVA was chosen for comparing group means, and t-tests were applied *post-hoc* if needed. All analyses were conducted using SPSS software, and Bonferroni or Holm corrections were applied to reduce Type I errors. Covariates were not included, as groups were balanced, but we are open to revising the model if future analysis suggests otherwise.

## 3 Results

In the results, the *f*EI ratio is calculated for each electrode of each subject across all EEG frequency bands within the four groups. However, no significant differences in *f*EI ratio between the different groups are found in the delta (0.5-4 Hz), theta (4-8 Hz), high beta (19-30 Hz), and gamma (> 30 Hz) bands, so our further discussion is confined to alpha (8-12 Hz) and lower beta (13-18 Hz) bands.

### 3.1 The *f*EI ratio in the alpha band

In this part of the study, the EEG signals of the control and SAD subjects are filtered to isolate the alpha band, and the fEI ratio is calculated over 5-second intervals for the 29 electrodes of each subject. Initially, we collectively analyzed the differences in the averaged *f*EI values between the four groups. To achieve this, the average *f*EI ratio for each subject is first calculated, followed by the calculation of the average *f*EI value within each group. The results indicate that the control group has a mean *f*EI value of 0.9883 ± 0.0846, suggesting balanced excitatory and inhibitory neural activities in the alpha band, which aligns with findings in (41). The mild SAD group, however, shows a slightly lower average ratio of 0.9550 ± 0.0716. In contrast, the moderate and severe SAD groups exhibit higher average *f*EI ratios of 1.0380 ± 0.0660 and 1.0017 ± 0.0835, respectively.

**Figure 2:**
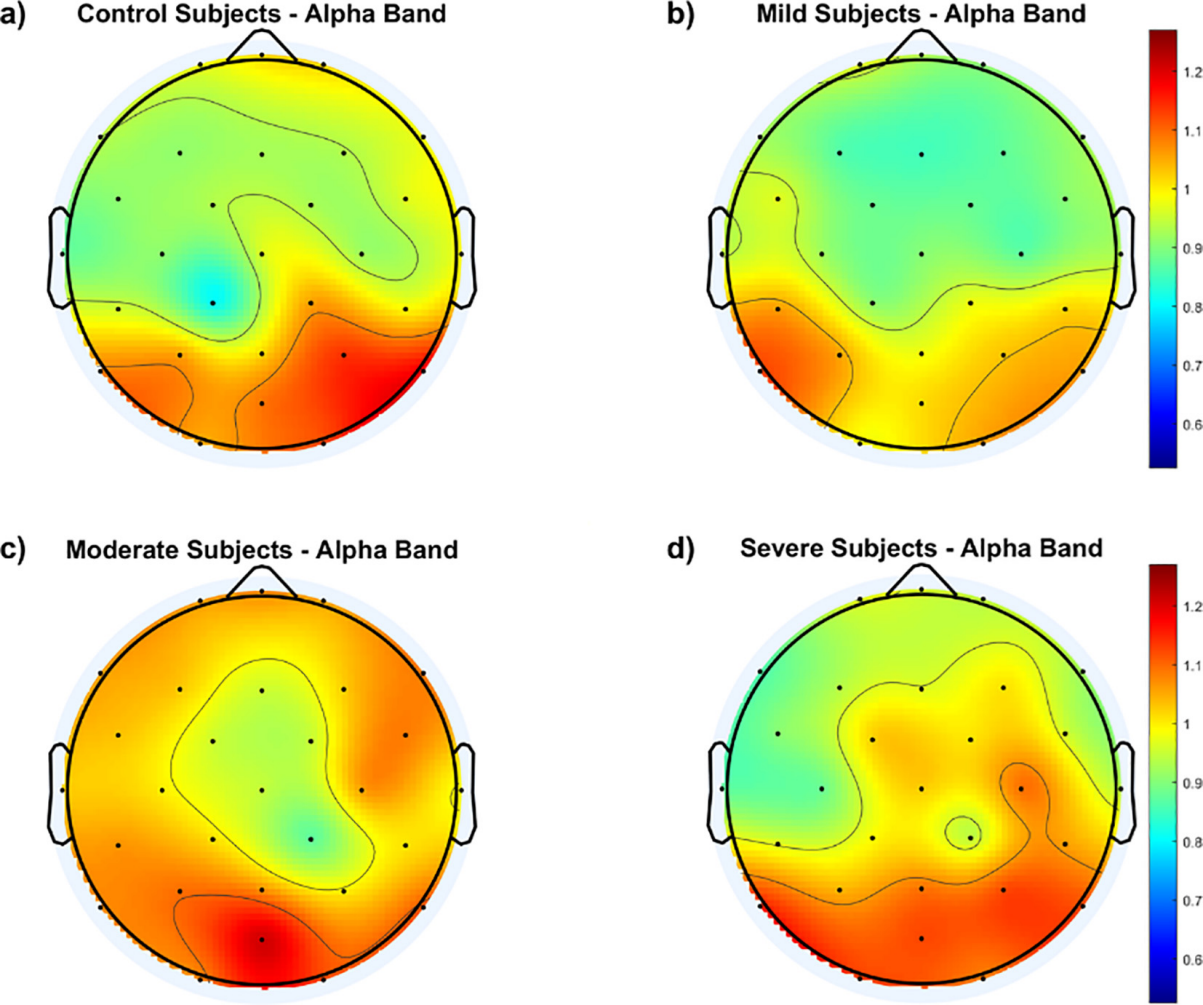
Topological maps of functional excitation-inhibition (fEI) ratios in the alpha band across different subject groups: **(A)** Control Subjects, **(B)** Mild Subjects, **(C)** Moderate Subjects, and **(D)** Severe Subjects. The color scale represents the intensity of fEI ratios, from low (blue) to high (red).

To gain further insight into the group differences, the average *f*EI within each group is calculated over the 29 electrodes and presented as a topographic map in **Figure 2**.

The results in **Figure 2** generally show higher *f*EI ratio values over the posterior regions of the cortex in all groups, including the control group. However, the control and mild groups display an inhibition-dominated network in the frontal, prefrontal, and central areas. In contrast, the moderate group exhibits an excitation-dominated network, with the severe group showing extended excitation dominance into the fronto-central region.

**Figure 3:**
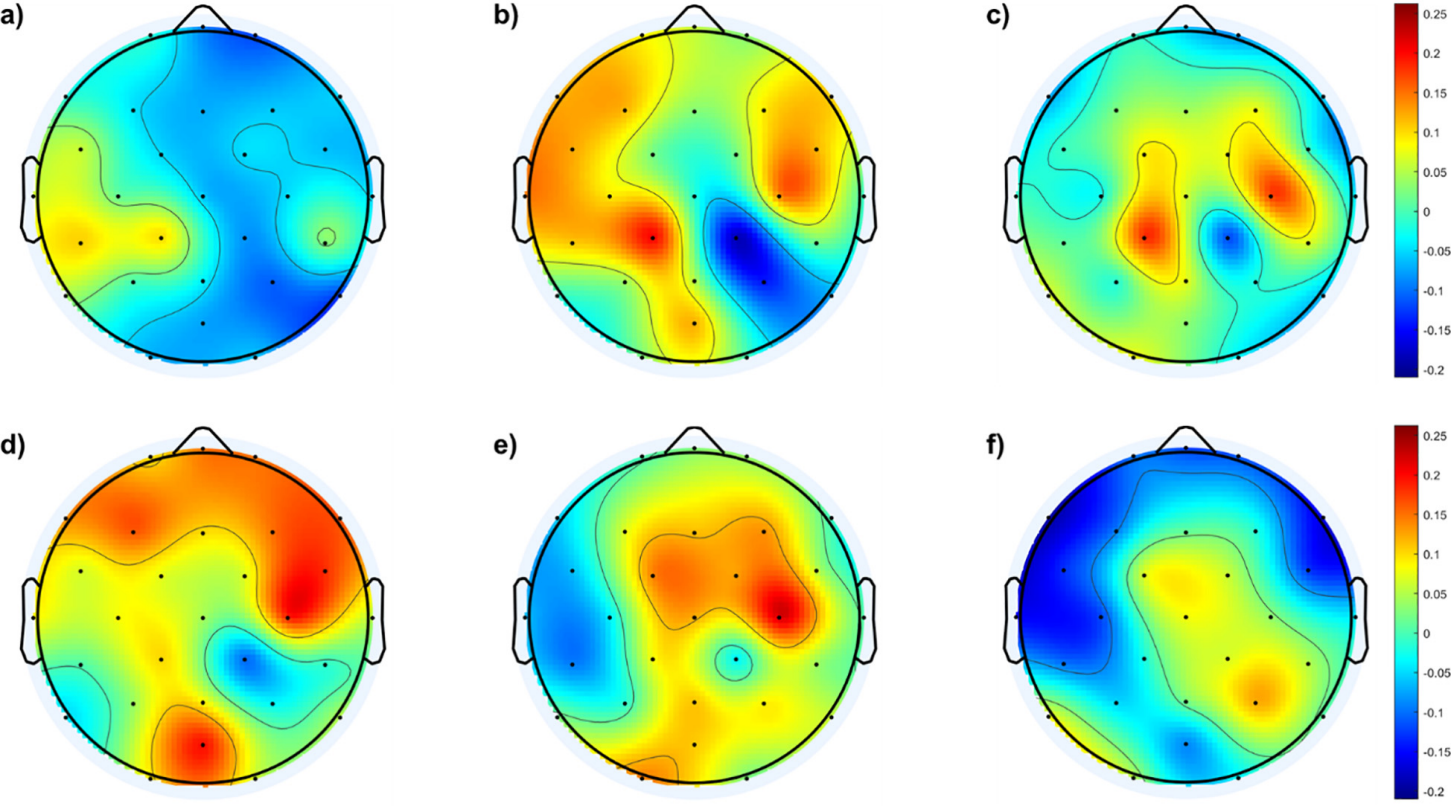
Topological maps of *f*EI differences in the alpha band between SAD and control groups and within the SAD groups: **(A)** shows the differences between individuals with mild SAD and healthy control subjects. **(B)** highlights the differences between individuals with moderate SAD and healthy control subjects. **(C)** illustrates the differences between individuals with severe SAD and healthy control subjects. **(D)** displays the differences between individuals with moderate SAD and those with mild SAD. **(E)** depicts the differences between individuals with severe SAD and those with mild SAD. Finally, panel **(F)** presents the differences between individuals with severe SAD and those with moderate SAD.

To further understand the differences in cortical distribution of the *f*EI values between the SAD groups and the control group, difference topographic maps are calculated and presented in **Figure 3**.

**Figure 3A** shows no significant differences between the mild and control groups across the cortical region, except for a mild increase in excitation in the left temporal and lateral-parietal regions of the mild group. In contrast, **Figure 3B** indicates higher *f*EI values in the lateral regions of both hemispheres when comparing the moderate group with the control group. **Figure 3C** reveals excessive excitation in the left and right central regions of the severe group compared to the control group.

In addition to comparisons with the control group, **Figure 3** also illustrates comparisons between different SAD groups. **Figure 3D** shows the *f*EI topographic differences in the alpha band between the mild and moderate groups, indicating excessive excitation in the frontal region of the moderate group compared to the mild group. **Figure 3E** demonstrates excessive excitation in the central area of the severe group compared to the mild group. Lastly, **Figure 3F** shows excessive inhibition in the frontal region and slightly higher excitation in the central and right parietal areas of the severe group compared to the moderate group.

To statistically address the differences in *f*EI values between the groups, a one-way ANOVA test was conducted at the electrode level. The test results show a significant difference in fEI values at electrode F7 between the moderate and severe groups (p = 0.0155, 95% CI = [0.02814, 0.3604]). Additionally, significant differences were found at electrode C4 between the mild and moderate groups (p = 0.0378, 95% CI = [-0.3840, -0.0080]) and between the mild and severe groups (p = 0.0239, 95% CI = [-0.4266, -0.0220]). Moreover, control subjects exhibited statistically significant differences compared to the moderate (p = 0.0081, 95% CI = [-0.3548, -0.0400]) and severe (p = 0.0242, 95% CI = [-0.3497, -0.0177]) groups at electrode CP1.

### 3.2 The fEI ratio in the low beta band

Previous EEG research indicates an inverse correlation between GABA neurotransmission and lower beta brainwaves, where higher GABA activity typically corresponds to reduced beta activity (42). Building on this, our study examines how neural activity in the lower beta band, which has been associated with GABAergic processes, relates to the fEI ratio. EEG data from control and SAD subjects are filtered to isolate the lower beta band, and fEI ratios are computed across 29 electrodes per subject. Group averages are then compared.

Results show similar mean *f*EI ratios between control (0.8521 ± 0.1016) and mild (0.8407 ± 0.0664) groups, but notably higher values are found in the moderate (0.9243 ± 0.0574) and severe (0.9234 ± 0.0607) SAD groups. ANOVA and *post hoc* tests revealed no significant difference between the control and mild groups. However, significant differences were observed between the moderate and severe SAD groups compared to controls. Specifically, the moderate group differed significantly from controls (p = 0.0017, 95% CI = [-0.1226 to -0.02169]), as did the severe group (p = 0.0020, 95% CI = [-0.1218 to -0.02087]). Comparing SAD groups, significant differences were noted between the mild group and both the moderate (p = 0.0002, 95% CI = [-0.1340 to -0.03308]) and severe (p = 0.0002, 95% CI = [-0.1332 to -0.03226]) groups.

These findings underscore the potential of the fEI ratio in the lower beta band as a biomarker not only for detecting SAD but also for assessing its severity.

**Figure 4:**
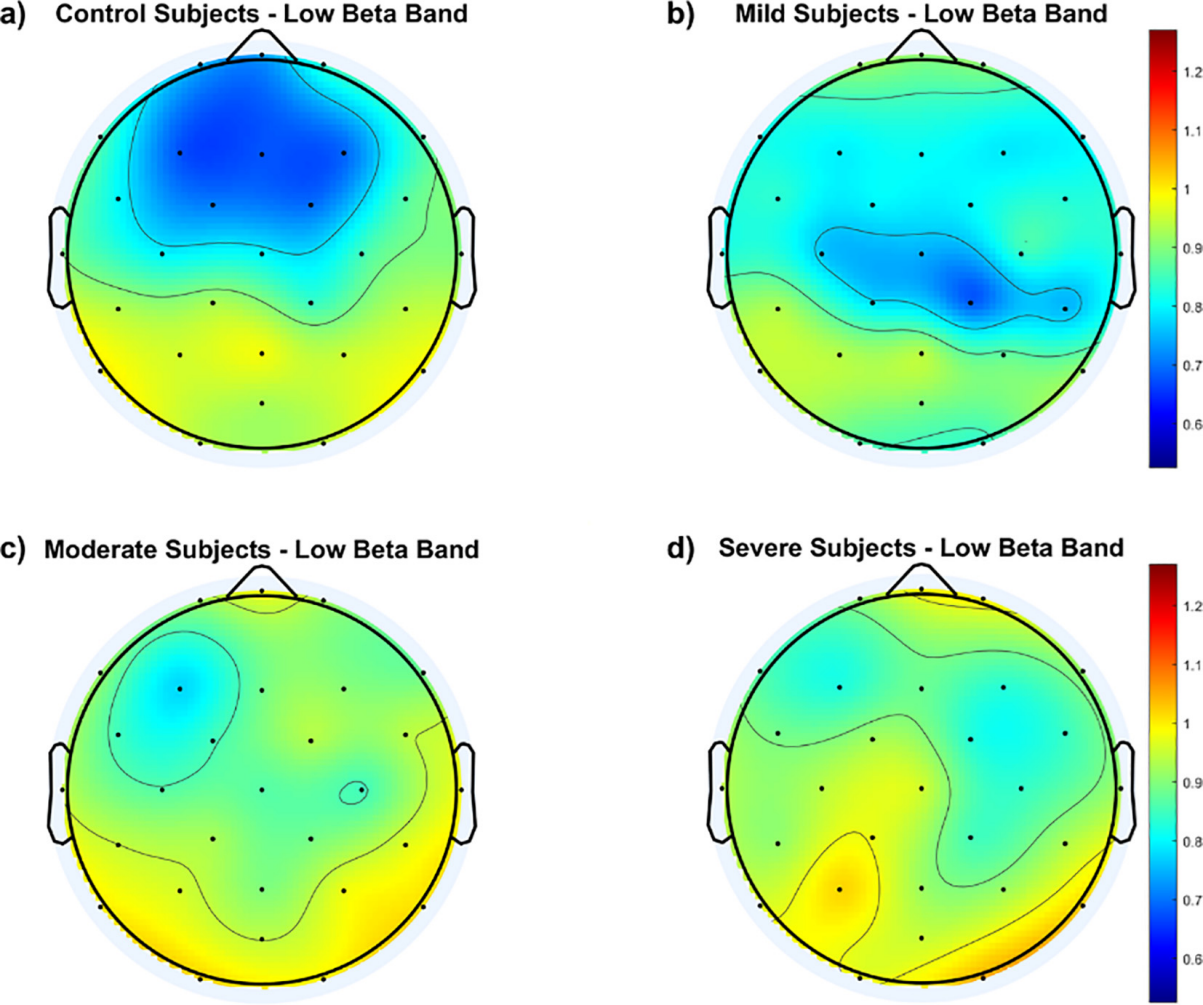
Topological maps of functional excitation-inhibition (fEI) ratios in the low beta band across different subject groups: **(A)** Control Subjects, **(B)** Mild Subjects, **(C)** Moderate Subjects, and **(D)** Severe Subjects. The color scale represents the intensity of fEI ratios, from low (blue) to high (red).

To illustrate variations in the *f*EI ratio within the low beta band across cortical regions, **Figure 4** displays topographic maps for all groups. Findings reveal notable distinctions between the control group and SAD groups across the frontal and central cortex. The control group exhibits an inhibition-dominated network in these regions, while SAD groups tend towards a more balanced network involving both inhibition and excitation.

Within the SAD groups, **Figure 4** indicates that the mild group maintains a balanced network in the fronto-central region, albeit to a lesser extent than the control group. In contrast, both the moderate and severe groups show significant departure from the inhibition-dominated network in the fronto-central cortex.

**Figure 5:**
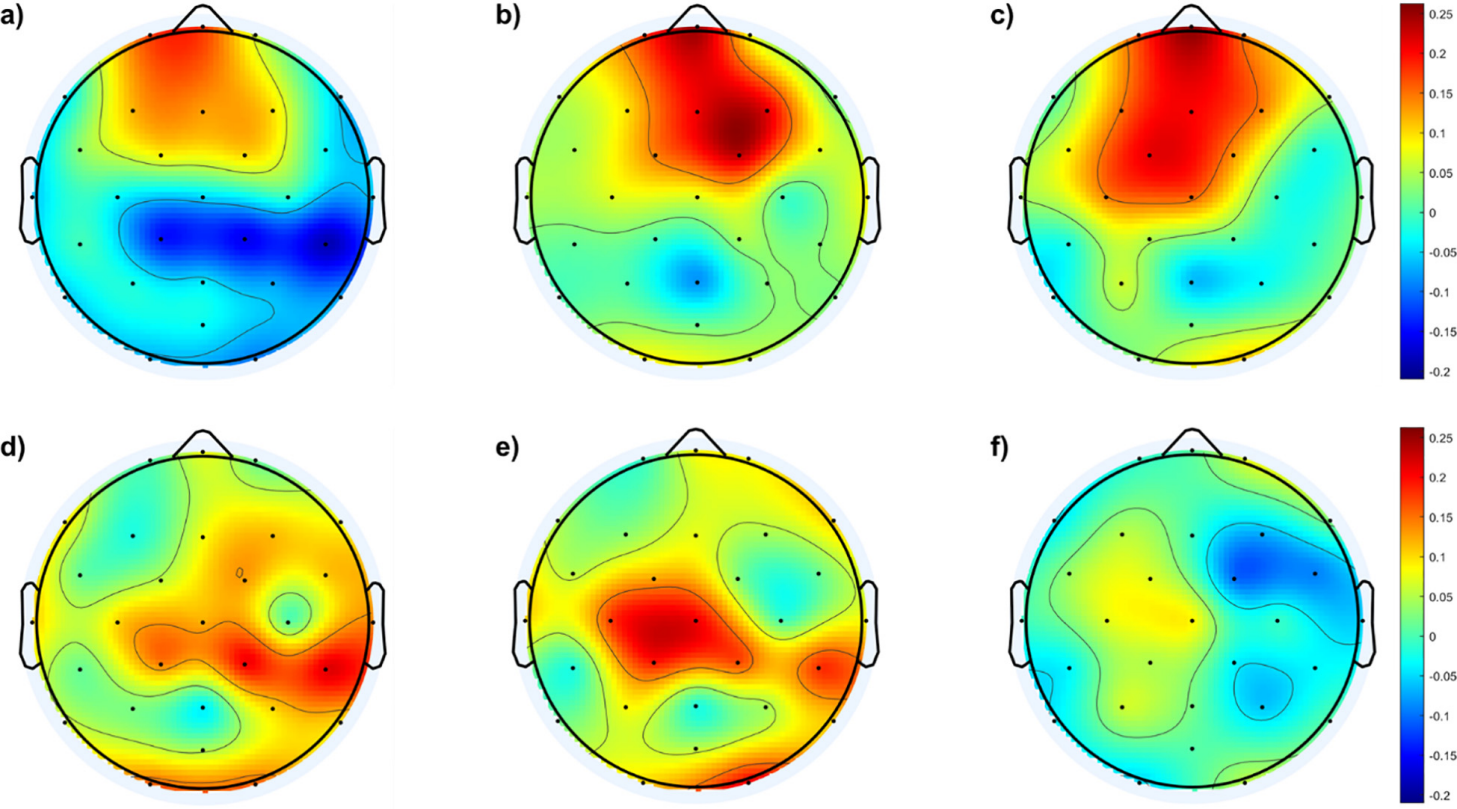
illustrates the topographic maps depicting differences in the *f*EI ratio among the four groups within the low beta band: **(A)** Difference between mild and control groups, **(B)** Difference between moderate and control groups, **(C)** Difference between severe and control groups, **(D)** Difference between moderate and mild groups, **(E)** Difference between severe and mild groups, **(F)** Difference between severe and moderate groups.

To further illustrate *f*EI ratio differences within the low beta band among the four groups, **Figure 5** depicts difference topographic maps. **Figures 5A–C** highlight fewer differences between the control and mild groups in the fronto-central region compared to differences with the other two groups. Conversely, **Figures 5D, E** reveal larger discrepancies between the moderate and severe groups compared to the mild group, particularly in the central-parietal cortex. **Figure 5F** indicates minimal differences between the moderate and severe groups.

To assess significant differences among electrodes in the SAD groups within the lower beta band, one-way ANOVA and subsequent *post hoc* tests are conducted. **Table 2** summarizes the results of the *post hoc* analysis, highlighting electrodes where significant differences are observed.

**Table 2:** Adjusted *p*-value of the *post hoc* test of low beta band.

Groups	Severe (N = 21)	Moderate (N = 22)	Mild (N = 18)
**Control** **(N = 21)**	Fpz: p = 0.0041 [-0.4510, -0.06674] FC1: p = 0.0333 [-0.4151, -0.01234]	Fp1: p = 0.0042 [-0.4208, -0.06176] Fpz: p = 0.0048 [-0.4326, -0.06099] FC2: p = 0.0041 [-0.4123, -0.06065]	CP6: p = 0.0350 [0.009562, 0.3636]
**Mild** **(N = 18)**	Cz: p = 0.0107 [-0.3691, -0.03641] CP2: p = 0.0483 [-0.3410, -0.0008917] CP6: p = 0.0406 [-0.3554, -0.005609] O2: p = 0.0292 [-0.3865, -0.01509]	CP2: p = 0.0084 [-0.3730, -0.04185] CP6: p = 0.0084 [-0.3893, -0.04336]	NA

**Table 2** reveals significant variations between the control group and both the moderate and severe SAD groups in the fronto-central cortex, specifically at electrodes Fpz, Fp1, FC1, and FC2. Furthermore, significant differences are noted between the mild group and both the moderate and severe groups in the centro-parietal region.

### 3.3 SAD severity as a function of the cortical regions

After computing the fEI ratio for each electrode, we group electrodes into regions to assess statistically significant differences across four subject groups. The regions of interest include prefrontal (Fp1, Fpz, Fp2), frontal (F7, F3, Fz, F4, F8), frontal-central (FC5, FC1, FC2, FC6), central (C3, Cz, C4), temporal (T7, T8), central-parietal (CP5, CP1, CP2, CP6), parietal (P7, P3, Pz, P4, P8), and occipital (POz, O1, O2). The fEI is calculated in each region and compared between the four groups in the alpha and low beta bands.

In the alpha band, the results of the ANOVA test are summarized in **Table 3**. The results reveal significant differences in the prefrontal and frontal regions among the groups. *Post hoc* comparisons show significant differences in the prefrontal region, particularly between the control group and the mild (p = 0.0098, 95% CI = [0.02095, 0.1325]), moderate (p = 0.0490, 95% CI = [-0.1118, -0.0002386]), and severe (p = 0.0457, 95% CI = [0.001115, 0.1127]) groups.

**Table 3:** ANOVA results comparing fEI ratios across brain regions in control and SAD severity groups in Alpha band.

Electrodes group	Subject Group				p-value	F-value
	Control (N = 21)	Mild (N = 18)	Moderate (N = 22)	Severe (N = 21)		
Prefrontal	1.0117 ± 0.0284	0.9350 ± 0.0268	1.0677 ± 0.0122	0.9548 ± 0.0120	0.0002	23.64
Frontal	0.9383 ± 0.0268	0.8883 ± 0.0339	1.0322 ± 0.0497	0.9386 ± 0.0528	0.0006	10.13
Frontal-central	0.9348 ± 0.0259	0.9076 ± 0.0399	1.0061 ± 0.0648	0.9727 ± 0.0552	0.0657	3.130
Central	0.9307 ± 0.0326	0.8968 ± 0.0233	1.0034 ± 0.0609	1.0028 ± 0.1062	0.1834	2.065
Temporal	0.9347 ± 0.0773	0.9356 ± 0.0066	1.0050 ± 0.0329	0.9033 ± 0.0431	0.3136	1.646
Central-parietal	0.9499 ± 0.1056	0.9868 ± 0.0653	0.9880 ± 0.0794	0.9867 ± 0.0490	0.8730	0.2311
Parietal	1.0924 ± 0.0659	1.0494 ± 0.0527	1.0661 ± 0.0442	1.1008 ± 0.0402	0.3983	1.048
Occipital	1.0903 ± 0.0349	1.0205 ± 0.0376	1.1370 ± 0.0635	1.1119 ± 0.0229	0.0470	4.176

This reflects a bidirectional GABAergic model, where the moderate group exhibits an excitation-dominated network (inhibition deficit model), and the severe group displays a balanced regime with heightened excitation in the occipital region and increased inhibition in the prefrontal cortex (over-inhibition model). The close values of fEI ratios between the control and severe groups suggest a homeostatic plasticity mechanism, where the prefrontal cortex has intensified inhibitory control to counter past overexcitation.

The frontal alpha asymmetry, known as a biomarker for anxiety, distinguishes the control and severe groups, with higher cortical activity in the right hemisphere indicating greater activity compared to the left frontal region in the severe group, associated with negative emotions.

The p-value column in **Table 3** refers to the significance of the differences between the four groups as a function of the cortical region. **Table 3** reveals significant differences between the four groups in the prefrontal (p = 0.0002) and frontal (p = 0.0006) regions in the alpha band.

In the low beta band, the variation in the fEI ratio across different cortical regions is shown in **Table 4**. The results show significant differences in all regions except for the parietal (p = 0.1156) and occipital (p = 0.1188) regions.

**Table 4:** ANOVA results comparing fEI ratios across brain regions in control and SAD severity groups in Beta band.

Electrodes group	Subject Group				p-value	F-value
	Control (N = 21)	Mild (N = 18)	Moderate (N = 22)	Severe (N = 21)		
Prefrontal	0.7767 ± 0.0646	0.9119 ± 0.0054	0.9444 ± 0.0339	0.9699 ± 0.0455	0.0025	11.97
Frontal	0.7553 ± 0.1003	0.8034 ± 0.0121	0.8730 ± 0.0550	0.8658 ± 0.0377	0.0221	4.233
Frontal-central	0.7673 ± 0.0809	0.8165 ± 0.0171	0.8931 ± 0.0496	0.8702 ± 0.0412	0.0227	4.622
Central	0.8118 ± 0.0364	0.7856 ± 0.0555	0.8621 ± 0.0069	0.9109 ± 0.0594	0.0369	4.630
Temporal	0.8946 ± 0.0071	0.8356 ± 0.0092	0.9586 ± 0.0315	0.9292 ± 0.0058	0.0077	19.25
Central-parietal	0.9071 ± 0.0449	0.7832 ± 0.1031	0.9307 ± 0.0335	0.9172 ± 0.0498	0.0461	8.048
Parietal	0.9749 ± 0.0219	0.9172 ± 0.0293	0.9696 ± 0.0466	0.9670 ± 0.0525	0.1156	2.306
Occipital	0.9359 ± 0.0053	0.8717 ± 0.0266	0.9862 ± 0.0397	0.9882 ± 0.0532	0.0125	7.012

Tukey’s HSD Test is used for multiple comparisons to identify which subject group’s mean fEI ratio significantly differs within each electrode group. The prefrontal and frontal regions are of particular interest.

In the prefrontal region, the control group shows statistically significant differences compared to all three severity groups (mild: p = 0.0205, 95% CI = [-0.2479, -0.02259]; moderate: p = 0.0062, 95% CI = [-0.2804, -0.05508]; severe: p = 0.0026, 95% CI = [-0.3058, -0.08058]). In the frontal region, while the mild group does not show significant differences compared to other groups, significant differences are observed between the control group and the moderate group (p = 0.0329, 95% CI = [-0.2272, -0.008269]) and between the control group and the severe group (p = 0.0474, 95% CI = [-0.2200, -0.001062]).

A clear trend of increased excitation in the prefrontal and frontal regions is noted in more severe SAD groups, correlating with higher fEI ratios. This aligns with the concept of inhibition deficits leading to overexcitation described in the bidirectional GABAergic model. Studies consistently report reduced GABA levels in the prefrontal cortex (PFC) of individuals with anxiety disorders, contributing to hyperexcitation. This hyperexcitation allows irrelevant or negative stimuli to overwhelm brain circuitry, intensifying anxiety. In the mild group, heightened PFC activity may reflect an initial overexcitation in response to stress. In the moderate and severe groups, this pattern extends to the frontal region, where increased excitation becomes more pronounced, potentially reflecting the cumulative effects of ongoing stress.

### 3.4 The whole brain *f*EI ratio

**Figure 6:**
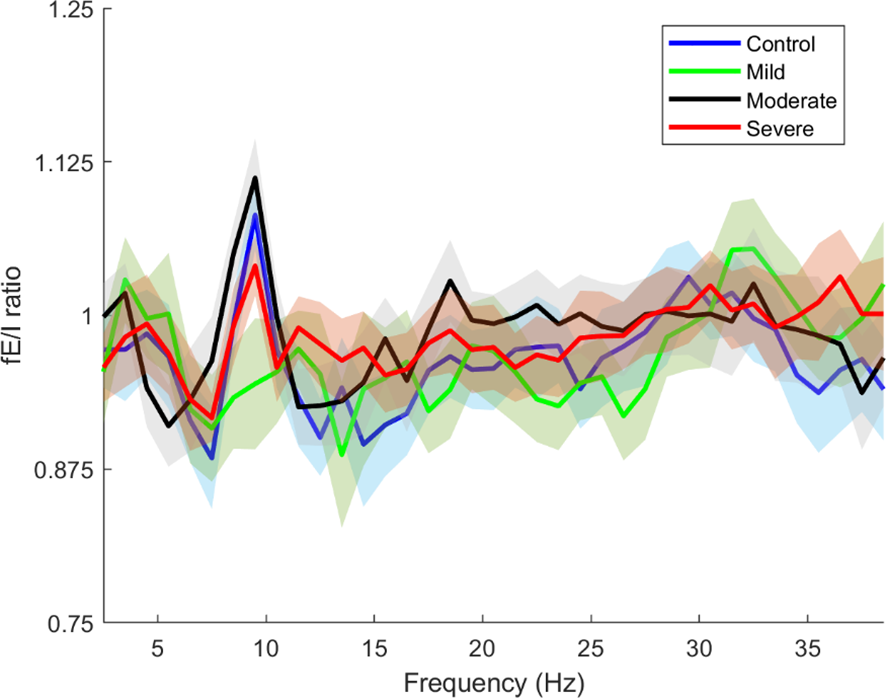
Whole brain functional excitation/inhibition (fE/I) of each severity estimated in the frequencies 2.5-38.5. The blue line is the whole brain fEI of the Control group, the green line is the whole brain fEI of the Mild group, the black line is the whole brain fEI of the Moderate group, the red line is the whole brain fEI of the Severe group. The solid lines and intervals represent mean and standard deviation values, respectively.

In this part of the results, we examined the overall changes in the *f*EI ratio across the entire cortex as a function of frequency. The total *f*EI ratio was computed for each severity group within the frequency range from 2.5 Hz to 38.5 Hz, with calculations performed at intervals of 2 Hz. **Figure 6** presents these findings, where solid lines indicate the average *f*EI values for each severity group, and shaded areas represent the corresponding standard deviations.

**Figure 6** clearly illustrates that the control and severity groups (excluding the mild group) exhibit peaks in the alpha band around 10 Hz, albeit with varying amplitudes. These peaks represent the mu frequency within the alpha band and can be attributed to several underlying physiological and functional mechanisms and reflect complex dynamics of neural activity and cortical processing (41, 48).

## 4 Discussion

### 4.1 Alpha and lower beta bands: key to fEI dynamics in SAD

In the developed results, significant differences in *f*EI ratio between the different groups are only found in alpha and lower beta bands. This finding is reasonable for several reasons. Firstly, these frequency bands are known to be relevant to cognitive and emotional processes implicated in anxiety disorders. The alpha band, typically associated with relaxed wakefulness and attentional modulation, showed alterations in fEI ratios that correlated with the severity of SAD. This aligns with previous research suggesting a link between alpha oscillations and states of heightened anxiety or cognitive control (41). Secondly, the lower beta band is involved in motor control, sensorimotor integration, and potentially in emotional regulation processes relevant to anxiety disorders. The observed patterns of increased fEI ratios in moderate and severe SAD groups within this band suggest dysregulated neural excitability, which could contribute to behavioral symptoms associated with anxiety.

Conversely, the absence of significant differences in the delta, theta, high beta, and gamma bands indicates that neural dynamics in these frequency ranges may not be as prominently altered in SAD, at least in the context studied. This specificity highlights the importance of frequency band analysis in understanding the nuanced neural correlates of anxiety disorders, focusing efforts on where meaningful differences in neural activity are detected.

In conclusion, while the study comprehensively analyzed the fEI ratio across multiple EEG frequency bands, the decision to concentrate on the alpha and lower beta bands for discussion reflects the relevance of these frequency ranges to the pathophysiology of SAD. This approach provides a clearer understanding of how neural network dynamics in these bands may contribute to the manifestation and severity of anxiety symptoms, offering insights into potential targets for future research and therapeutic interventions.

### 4.2 fEI dynamics in the alpha band for SAD

In Section 3.1, the results focused on the alpha band, in exploring fEI ratio across different severity groups of SAD compared to controls. The findings revealed distinct patterns in fEI ratios across the severity spectrum of SAD. The control group exhibited a mean fEI ratio of 0.9883 ± 0.0846, indicating a balanced state of neural excitability and inhibition within the alpha band. This balanced ratio suggests that healthy individuals maintain efficient regulatory mechanisms that stabilize cortical activity, supporting normal cognitive functions and emotional regulation (41). Conversely, individuals with mild SAD showed a slightly lower average fEI ratio of 0.9550 ± 0.0716. This reduction suggests a subtle shift towards increased inhibition relative to excitation, which may reflect initial compensatory mechanisms in response to anxiety-related stimuli. This finding aligns with theories suggesting that early stages of anxiety disorders involve heightened regulatory efforts to manage emotional arousal and cognitive control (45).

The most notable observations were in the moderate and severe SAD groups, where fEI ratios were notably higher at 1.0380 ± 0.0660 and 1.0017 ± 0.0835, respectively. These elevated ratios indicate a pronounced imbalance towards increased neural excitation within the alpha band. Such dysregulation may signify compromised inhibitory control mechanisms, potentially contributing to heightened sensitivity to stressors and exaggerated emotional responses characteristic of moderate to severe anxiety states (46).

From a physiological perspective, the alpha band is crucial for orchestrating neural synchronization and communication across cortical regions involved in attentional processes and emotional regulation. The observed deviations in fEI ratios across SAD severity groups suggest disruptions in these essential functions. Increased excitation in the moderate and severe SAD groups could reflect maladaptive changes in GABAergic inhibition, a neurotransmitter system critical for maintaining cortical stability and preventing hyperexcitability (47).

Overall, these findings underscore the relevance of the fEI ratio as a biomarker for understanding the neural underpinnings of SAD. The shifts in neural excitability and inhibition within the alpha band provide insights into how cortical dynamics are altered in anxiety disorders, informing potential avenues for therapeutic interventions aimed at restoring EI balance and improving cognitive-emotional functioning in affected individuals.

Continuing from this analysis of fEI ratios in the alpha band, the topographic maps presented in **Figures 2**, **3** provide deeper insights into the spatial distribution of neural excitation-inhibition dynamics across different severity groups of SAD compared to controls. **Figure 2** illustrates that while all groups, including controls, exhibit higher fEI ratio values over posterior cortical regions, significant differences emerge in frontal and fronto-central areas among the SAD groups. Specifically, the control and mild SAD groups exhibit an inhibition-dominated pattern in the frontal, prefrontal, and central areas, indicative of effective regulatory mechanisms maintaining neural balance in these regions. In contrast, the moderate SAD group shows a notable shift towards an excitation-dominated network, particularly evident in frontal areas. This suggests compromised inhibitory control mechanisms, potentially contributing to heightened neural activity and emotional dysregulation associated with moderate anxiety states. Similarly, the severe SAD group extends this excitation dominance into the fronto-central region, indicating further dysregulation and potentially reflecting more severe symptoms of anxiety impacting broader cortical regions involved in emotional processing and cognitive control.

**Figure 3** complements these findings by presenting difference topographic maps that highlight significant contrasts between the SAD groups and controls, as well as within different severity levels of SAD. **Figures 3A–C** illustrate these differences succinctly: while **Figure 3A** shows minimal differences between the mild SAD and control groups, except for slight increases in specific regions, **Figures 3B, C** clearly depict increased fEI values in lateral and central regions of the brain in moderate and severe SAD groups compared to controls, respectively.

Furthermore, comparisons within the SAD groups (**Figures 3D-F**) reveal distinct regional patterns. **Figure 3D** indicates excessive excitation in the frontal region of the moderate SAD group compared to the mild group, suggesting a progressive dysregulation of cortical excitability with increasing severity. **Figure 3E** shows heightened excitation in central areas of the severe SAD group compared to the mild group, highlighting pronounced alterations in neural dynamics across different brain regions. Lastly, **Figure 3F** reveals a complex pattern in the severe SAD group, characterized by excessive inhibition in frontal regions alongside increased excitation in central and right parietal areas compared to the moderate group.

Statistically, the one-way ANOVA tests conducted at the electrode level confirm these observations, identifying specific electrodes where significant differences in fEI values occur between groups. For instance, significant differences at electrodes F7, C4, and CP1 underscore the regional specificity of these neural alterations across different severities of SAD compared to controls. These findings collectively suggest that alterations in fEI ratios within the alpha band are not only indicative of neural dysregulation in anxiety disorders but also highlight specific cortical regions where these dysregulations manifest prominently.

In conclusion, the spatial distribution of fEI ratios provides critical insights into the neurophysiological underpinnings of SAD, reflecting dysregulated neural dynamics across frontal, central, and posterior cortical regions. These findings contribute to our understanding of how anxiety disorders alter neural processing, offering potential targets for future research and therapeutic interventions aimed at restoring neural balance and improving clinical outcomes in affected individuals.

### 4.3 fEI dynamics in the lower beta band for SAD

In Section 3.2, the study investigates how GABAergic activity in the lower beta band correlates with the fEI ratio across cortical regions in individuals with Social Anxiety Disorder (SAD) compared to controls, focusing on physiological implications. The findings reveal that while the control and mild SAD groups exhibit similar mean fEI ratios, indicative of balanced excitatory and inhibitory neural activities in the lower beta band, the moderate and severe SAD groups display significantly higher fEI ratios. This suggests an imbalance characterized by increased neural excitability relative to inhibition in more severe forms of SAD.

Physiologically, elevated fEI ratios in the moderate and severe SAD groups likely reflect dysregulated GABAergic inhibition. GABA, the primary inhibitory neurotransmitter in the brain, regulates neural excitability, and higher fEI ratios in SAD may indicate reduced GABAergic tone or impaired inhibitory control mechanisms. The observed shift towards excitation dominance in the frontal and fronto-central regions of the brain in moderate and severe SAD aligns with theories implicating GABA deficits in anxiety disorders. This dysregulation may contribute to heightened neural responsiveness to stressors and exaggerated emotional responses characteristic of SAD.

The topographic maps in **Figure 4** underscore these physiological insights by illustrating regional differences in fEI ratios across groups. Control subjects show a typical inhibition-dominated network in frontal and central areas, indicative of healthy regulatory processes. In contrast, SAD groups exhibit a more balanced network between excitation and inhibition, suggesting compromised inhibitory control. This pattern intensifies in the moderate and severe groups, where excessive excitation spreads into fronto-central regions, potentially amplifying anxiety-related neural responses.

**Figure 5** further supports these physiological interpretations by highlighting how regional variations in fEI ratios differ among the control and SAD groups within the lower beta band. The minimal differences between control and mild SAD groups suggest relatively preserved inhibitory function in milder anxiety states. Conversely, significant deviations observed between moderate and severe SAD groups compared to controls, particularly in central-parietal regions, indicate progressive neural dysregulation across severity levels.

Statistical analyses in **Table 2** confirm these physiological insights by pinpointing specific electrodes where significant differences in fEI ratios occur. Notably, differences in frontal and fronto-central regions underscore the localized impact of dysregulated neural inhibition in SAD. Furthermore, the observed variations in centro-parietal regions between mild and severe SAD groups highlight the dynamic nature of neural alterations in anxiety disorders, suggesting a continuum of GABAergic dysfunction corresponding to disease severity.

In conclusion, the study provides physiological evidence linking altered GABAergic modulation and elevated fEI ratios in the lower beta band to disrupted neural network dynamics in SAD. These findings deepen our understanding of the neurophysiological mechanisms underpinning anxiety disorders, emphasizing the potential of the fEI ratio as a biomarker for assessing disease severity and informing targeted therapeutic strategies aimed at restoring neural balance and improving clinical outcomes.

### 4.4 Neurophysiological insights into GABAergic dysregulation and fEI ratios in SAD

The results in Sections 3.3 shed light on the neurophysiological underpinnings of SAD. In the alpha band, significant differences observed in the prefrontal and frontal regions among the control and SAD groups underscore dysregulations in GABAergic neurotransmission, pivotal for inhibitory control in anxiety states. The control group demonstrates a balanced inhibitory network across the prefrontal and frontal areas, consistent with regulatory processes supporting emotional regulation and cognitive function. In contrast, the moderate and severe SAD groups exhibit disrupted neural dynamics characterized by elevated fEI ratios, indicating heightened neural excitability relative to inhibition. This imbalance suggests compromised GABAergic function, corroborating theories of inhibition deficits contributing to overexcitation in anxiety disorders (43, 45–47).

**Table 3** represents these findings, highlighting similarities between the control and severe groups, where the latter shows intensified inhibitory control in the prefrontal cortex, potentially as a compensatory response to past overexcitation. This observation aligns with homeostatic plasticity mechanisms implicated in anxiety states, where neural circuits attempt to restore balance amidst chronic stress and dysregulation (43). Moreover, frontal alpha asymmetry, an established biomarker for anxiety (44), distinguishes these groups, with increased right hemispheric activity in the severe group indicative of heightened emotional processing and vigilance to threat stimuli.

In the low beta band, significant differences across most regions further elucidate the regional specificity of neural dysregulation in SAD. The prefrontal and frontal regions, crucial for cognitive and emotional processing, exhibit pronounced excitation in more severe SAD groups, as indicated by higher fEI ratios. This progression of increased excitation correlates with disease severity and supports the utility of fEI ratios as biomarkers for assessing SAD severity. The findings underscore the clinical relevance of understanding GABAergic dysregulation in anxiety disorders, suggesting potential targets for therapeutic interventions aimed at restoring neural balance and alleviating symptoms associated with SAD.

In **Figure 6**, the observed peaks in the alpha band around 10 Hz (mu frequency) among the control and severity groups (excluding the mild group) underscore significant neurophysiological insights into SAD. These findings suggest distinct patterns of neural excitation and inhibition dynamics across different SAD severity levels, with implications for understanding cortical processing in anxiety states.

The mu frequency range is associated with the mirror neuron system (MNS), which is involved in understanding the actions and emotions of others by internally simulating them (49). Abnormalities in MNS functioning have been linked to social and emotional processing deficits observed in conditions like SAD and autism spectrum disorders (50). Our results show an elevated fE/I ratio at mu frequencies in SAD subjects, especially in the mild group, suggesting an altered excitation/inhibition balance in the neural circuits underlying the MNS. The decrease in mu rhythm synchronization is believed to indicate the integration of motor functions (51) along with the activation of the MNS (52). The higher fE/I ratio observed at mu frequencies in SAD may suggest changes in how sensory and motor information are processed and combined with emotional cues, leading to challenges in responding during interactions due to a lack of seamless integration between sensory input and motor responses. In the group with milder symptoms, a peak at 11.5 Hz hints at a subtle adjustment in the optimal frequency for sensorimotor integration.

Additionally, despite experiencing heightened excitation, the severe SAD group shows an fE/I ratio at mu frequencies that is more similar to that of the control group. This could indicate the presence of compensatory mechanisms or homeostatic balancing at extreme levels of anxiety, reflecting an adaptation aimed at managing levels of anxiety and social discomfort. In this scenario, the brain may be striving to restore an excitation/inhibition ratio at mu frequencies to support the functioning of the MNS and sensorimotor integration.

Further research into these frequency-specific dynamics could elucidate mechanisms underlying SAD pathophysiology and inform targeted therapeutic interventions aimed at restoring neural balance and improving clinical outcomes.

## 5 Conclusion

This paper has explored the intricate neurobiological mechanisms underlying social anxiety disorder (SAD) through the lens of the functional excitation-inhibition (fEI) ratio measured via resting-state EEG. By investigating patterns of neural activity in the alpha and lower beta bands, our study aimed to uncover objective biomarkers for detecting and assessing the severity of SAD. The findings highlight significant alterations in the fEI ratio across different severity levels of SAD, particularly in the alpha and lower beta frequency bands. In the alpha band, individuals with moderate to severe SAD exhibited elevated fEI ratios, indicating heightened neural excitability relative to inhibition compared to healthy controls. These observations suggest compromised inhibitory control mechanisms, potentially contributing to the heightened emotional reactivity and cognitive dysregulation characteristic of SAD. Moreover, our study identified regional specificity in these neural dynamics, with frontal and fronto-central areas showing pronounced dysregulation in more severe cases of SAD. This regional pattern underscores the localized impact of neural dysfunction in areas crucial for emotion regulation and cognitive processing. The insights gained from this research underscore the potential of the fEI ratio as a biomarker for assessing SAD severity and understanding its neurophysiological underpinnings. By elucidating these mechanisms, future studies can explore targeted therapeutic interventions aimed at restoring neural balance and improving clinical outcomes for individuals affected by SAD.

## Data Availability

The original contributions presented in the study are included in the article/supplementary material. Further inquiries can be directed to the corresponding author.
